# The Association of Heart Failure and Liver T1 Mapping in Cardiac Magnetic Resonance Imaging

**DOI:** 10.3390/diagnostics15060779

**Published:** 2025-03-20

**Authors:** Adrian T. Huber, Joanna Bartkowiak, Robin Seitz, Benedikt Bernhard, Martina Boscolo Berto, Giancarlo Spano, Benedikt Wagner, Verena C. Obmann, Lukas Ebner, Inga A. S. Todorski, Michael P. Brönnimann, Kady Fischer, Dominik P. Guensch, Andreas Christe, Annalisa Berzigotti, Lorenz Räber, Tobias Reichlin, Thomas Pilgrim, Fabien Praz, Christoph Gräni, Nicholas Brugger, Alan A. Peters

**Affiliations:** 1 Department of Diagnostic, Interventional and Pediatric Radiology, Inselspital, Bern University Hospital, University of Bern, Rosenbühlgasse 27, 3010 Bern, Switzerland; 2 Radiology and Nuclear Medicine, Luzerner Kantonsspital, 6000 Luzern, Switzerland; 3 Department of Radiology, Beau-Site Hospital, Hirslanden Bern, 3013 Bern, Switzerland; 4 Department of Cardiology, Inselspital, Bern University Hospital, University of Bern, 3010 Bern, Switzerland; 5 Department of Radiology, Zuger Kantonsspital, 6340 Baar, Switzerland; 6 Department of Anesthesiology and Pain Medicine, Inselspital, Bern University Hospital, University of Bern, 3010 Bern, Switzerland; 7 Department of Hepatology, Inselspital, Bern University Hospital, University of Bern, 3010 Bern, Switzerland

**Keywords:** liver, heart failure, magnetic resonance imaging, cardiomyopathies, hepatic veins, extracellular space

## Abstract

**Background/Objectives:** The objective of this study was to investigate the association between congestive heart failure (CHF) and T1 mapping in both liver lobes using cardiac MRI. **Methods:** This retrospective study included patients who underwent cardiac MRI with T1 mapping sequences on a 1.5 T scanner. The liver T1 values were measured in four hepatic regions, utilizing cardiac short axis and four-chamber views. Echocardiographic and laboratory data were collected within 90 days of the cardiac MRI. Comparisons of the liver T1 values and echocardiographic parameters between patients with and without elevated NT-proBNP levels (>125 pg/mL) were conducted using the Mann–Whitney U test. Logistic regression models were employed to adjust for confounding factors. **Results:** A total of 397 patients were included (with a median age of 56 years; 127 females), of whom 35% (*n* = 138) exhibited elevated NT-proBNP levels. The patients with elevated NT-proBNP levels showed a larger end-diastolic volume (EDV: 92 vs. 81 mL/m^2^, *p* < 0.001) and a lower LVEF level (50% vs. 60%, *p* < 0.001). The liver T1 was significantly higher in the right liver lobe (670 vs. 596 ms, *p* < 0.001) and the caudate lobe (664 vs. 598 ms, *p* < 0.001), but not in the left lobe (571 vs. 568 ms, *p* = 0.068) or the dome (590 vs. 560 ms, *p* = 0.1). T1 mapping in the caudate (OR 1.013, 95% CI 1.004–1.023, *p* = 0.005) and right liver lobes (OR 1.012, 95% CI 1.003–1.021, *p* = 0.009) remained independently predictive in the logistic regression analysis. **Conclusions:** Elevated T1 values in the caudate and right liver lobes assessed by cardiac MRI were independently associated with CHF and outperformed T1 measurements in the left liver lobe in predicting disease.

## 1. Introduction

Heart failure is a common indication for cardiac magnetic resonance imaging (MRI) [1] and has been associated with poor cardiovascular outcomes in patients with myocardial infarction [2], non-ischemic cardiomyopathy [3,4], myocarditis [5], valvular heart disease [6], and pulmonary hypertension [7]. Heart failure is associated with elevated left ventricular filling pressures that may be estimated non-invasively by N-terminal B-type natriuretic peptide (NT-proBNP) [8,9] and diastolic evaluation using echocardiography [10,11]. While the diastolic parameters derived from cardiac MRI correlate well with echocardiographic measurements, their association with invasive filling pressure assessments remains moderate in both modalities [12,13]. A combination of the left atrial volume index (LAVI) with mitral flow and tissue Doppler (TDI) may increase the accuracy in echocardiography [14]. Similarly, cardiac MRI integrates functional parameters with myocardial T1, T2, and extracellular volume (ECV) mapping to evaluate myocardial fibrosis, edema [15,16], and outcome prediction [17]. However, a fast and straightforward parameter to identify elevated central venous pressure and congestion would significantly simplify the assessment of patients with suspected heart failure.

The liver, as a large upstream organ affected by cardiac function, is often involved in heart failure but is frequently overlooked in clinical evaluations [18]. Congestive liver disease, characterized by passive sinusoidal dilatation, can progress to peri-sinusoidal collagen deposition, leading to cardiac liver fibrosis and cirrhosis [19,20]. While cardiac cirrhosis is irreversible, cardiac liver congestion is transient and asymptomatic in most cases with absent or only mild liver fibrosis [21].

Recently, elevated ultrasound-based transient elastography liver stiffness measurements have been associated with elevated cardiac filling pressures and adverse outcomes in patients with heart failure [22]. Unfortunately, elastography techniques are not usually available during cardiac MRI or require additional hardware installation [23].

T1 mapping sequences are nowadays routinely performed during cardiac MRI and do not only cover the heart but also parts of the liver in the standard fields of view. T1 measurements of the liver are rapidly performed in a single region of interest (ROI) without the necessity for additional image acquisitions or post-processing and may yield important information about liver congestion. In a recent proof-of-concept cardiac MRI study, patients with decompensated dilated cardiomyopathy (DCM) had higher liver T1 values than patients with compensated DCM [24].

This study aimed to evaluate the association of magnetic resonance liver T1 mapping with congestive heart failure and its role as a non-invasive imaging biomarker.

## 2. Materials/Methods

### 2.1. Primary Study Population

This retrospective study included patients who underwent cardiac MRI between January 2017 and December 2019, incorporating T1 and T2 mapping sequences acquired on a single 1.5 T scanner. This study was approved by the local ethics committee and conducted in compliance with the principles outlined in the Declaration of Helsinki. Written informed consent was obtained from all the participants.

The exclusion criteria included ages below 18 years, a refusal to provide consent, or a history of chronic liver disease (CLD). The CMR indications of all the examinations are provided in the Supplementary Materials (Table S1).

### 2.2. Baseline Evaluation

Clinical and laboratory data were extracted from electronic medical records, while cardiac MRI information was obtained from the Centricity RIS system (GE Healthcare, General Electric Company, Chicago, IL, USA).

The laboratory parameters analyzed included liver markers (bilirubin, alanine aminotransferase [ALAT], and aspartate aminotransferase [ASAT]), cardiac biomarkers (troponin-T and NT-proBNP), and general indicators such as hematocrit and creatinine levels. Clinical data, including age, body mass index (BMI), and history of myocardial infarction, were also collected for all the participants (Table 1).

Heart failure was defined as an NT-proBNP level exceeding 125 pg/mL, consistent with the European Society of Cardiology (ESC) guidelines [25].

### 2.3. Cardiac Magnetic Resonance Imaging Protocol

All the participants underwent imaging on a Siemens MAGNETOM Aera 1.5 T scanner (Siemens Healthineers, Erlangen, Germany) using a 6-channel body coil. The standard cardiac MRI sequences were acquired during end-expiratory breath-holds, including steady-state free precession (SSFP) cine images in short axis, two-chamber, three-chamber, and four-chamber views. The imaging parameters included a field of view of 276 × 340 mm, a matrix size of 208 × 256, a repetition time/echo time (TR/TE) of 2.4/1.2 ms, an in-plane resolution of 1.4 × 1.4 mm, a section thickness of 8 mm, and a flip angle of 80°.

The T1 mapping was performed with motion correction using a modified Look–Locker inversion recovery sequence (5[3]3 scheme), acquired 15 min before and after the intravenous contrast administration. The imaging parameters for the T1 mapping included a TR/TE of 344/1.12 ms, a matrix size of 218 × 256, a flip angle of 35°, a pixel size of 1.41 × 1.41 mm, and a slice thickness of 8 mm.

The T2 mapping was conducted with motion correction using a three-point T2-prepared steady-state free precession sequence prior to the contrast administration. The parameters for the T2 mapping included TR/TE values of 300/0, 24, and 55 ms; a matrix size of 206 × 256; a flip angle of 35°; a pixel size of 1.41 × 1.41 mm; and a slice thickness of 8 mm.

### 2.4. Echocardiography Analysis

The echocardiography examinations were reviewed by two cardiology fellows (J.B. and B.B.) under the guidance of an echocardiography expert (N.B.). Echocardiograms performed within 90 days of the cardiac MRI were considered eligible for analysis. The measurements were conducted using TOMTEC-ARENA software (TTA 2.51, Tomtec Imaging GmbH, Unterschleissheim, Germany).

The left atrial end-systolic diameter and volume were measured from the parasternal long axis, apical four-chamber, and apical two-chamber views. The left ventricular diastolic function was assessed through a pulsed wave Doppler of the mitral inflow and tissue Doppler imaging (TDI) of the mitral annulus in the apical four-chamber view. Key metrics included the mitral inflow early-to-late diastolic flow ratio (E/A), peak early diastolic TDI velocity at the mitral annulus (mean E’), and the ratio of the early diastolic transmitral flow velocity to the mitral annular early diastolic TDI velocity (mitral E/E’).

The left ventricular systolic function was evaluated using the ejection fraction, calculated by Simpson’s biplane method. The right ventricular systolic function was assessed via the tricuspid annular plane systolic excursion (TAPSE), the peak systolic TDI velocity at the right ventricular lateral annulus (RV S’), and measurements of the right ventricular global and free wall longitudinal strain. The left ventricular filling pressures were analyzed following the European Association of Cardiovascular Imaging guidelines [26].

### 2.5. Cardiac MRI Analysis

The cardiac functional parameters and volumetric data were evaluated by the radiology and cardiology fellows (A.P., I.T., M.B., B.W., and G.S.) under the supervision of two experienced imaging specialists (C.G. and A.H.). The left and right ventricular epicardial and endocardial borders were segmented using a dedicated workstation (SyngoVia VB30, Siemens Healthcare, Erlangen, Germany). The regions of interest (ROIs) were manually delineated on the liver in the short axis and four-chamber views from the T1 and T2 mapping sequences (R.S. and A.P.), carefully avoiding the vascular and biliary structures.

**Figure 1:**
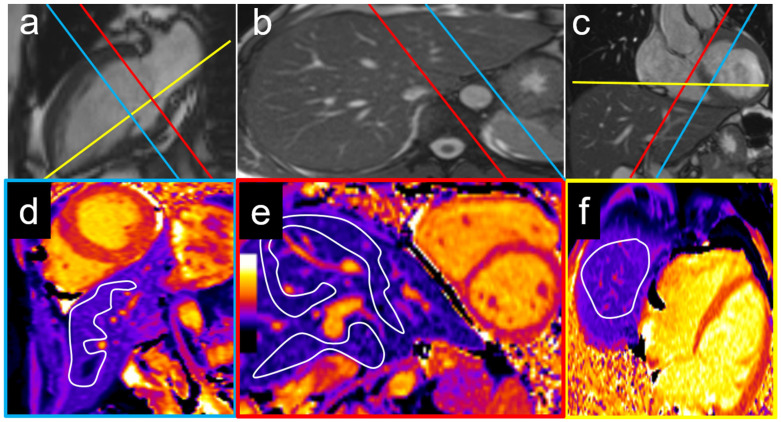
Examples of T1 measurement locations with corresponding cardiac two-chamber (**a**), axial (**b**), and coronal views (**c**). The left lobe was measured on a mid-ventricular short axis view (blue plane) with a T1 of 536 ms (**d**), while the right lobe and caudate lobe were measured on a basal short axis view (**e**) with T1 times of 583 ms and 614 ms, respectively (red plane). An exemplary measurement of the liver dome on a cardiac four-chamber view (yellow plane) is shown in (**f**) with a liver T1 of 534 ms.

The ROIs were placed in the following liver regions: the left liver lobe (segments II and III) on the cardiac midventricular short axis view, the right liver lobe (segments IVa and VIII) and caudate lobe (segment I) on the cardiac basal short axis view, and the liver dome (segment VIII) on the cardiac four-chamber view (Figure 1). The extracellular volume (ECV) was computed using the following formula:

$$ECV=\left(1-hematocrit\right)\frac{\frac{1}{post\;contrast\;T1\;liver}-\frac{1}{native\;T1\;liver}}{\frac{1}{post\;contrast\;T1\;blood}-\frac{1}{native\;T1\;blood}}$$

### 2.6. Statistical Analysis

The continuous variables are presented as medians with the interquartile ranges (IQRs), and the group comparisons were conducted using the Mann–Whitney U test. The categorical variables are expressed as absolute numbers and percentages, with the comparisons between the groups performed using Fisher’s exact test. To evaluate the predictive value of parameters such as T1 relaxation time, age, and gender for elevated NT-proBNP levels, a univariate (binary) logistic regression analysis was conducted. Subsequently, a multivariate logistic regression model was applied to account for potential interdependencies among significant parameters. A *p*-value of <0.05 was considered indicative of statistical significance. All the statistical analyses and data visualizations were carried out using SPSS Statistics (version 25.0, IBM Corp., Armonk, NY, USA) and GraphPad Prism (version 8, GraphPad Software, Inc., San Diego, CA, USA).

## 3. Results

### 3.1. Final Study Population

**Figure 2:**
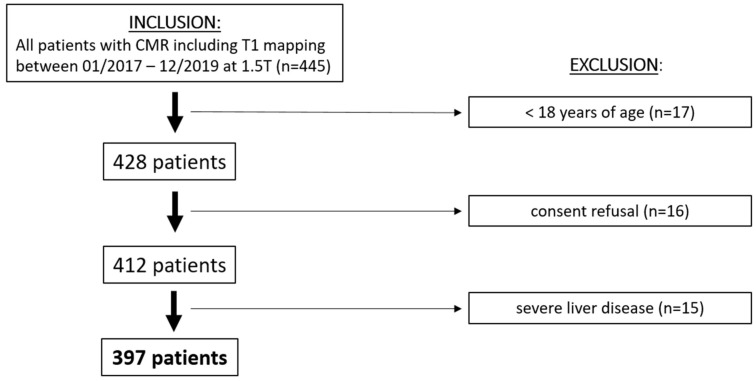
Patient flowchart. MRI = magnetic resonance imaging.

Among 447 consecutive patients who underwent cardiac MRI with T1 and T2 mapping between 01/2017 and 12/2019, 397 patients met the inclusion criteria (Figure 2, Table 1). The median age was 56 years [IQR, 37–69 years]. A total of 127 participants (32%) were females, and 138 participants (35%) presented with elevated NT-proBNP. The patients with elevated NT-proBNP were older (61 [IQR, 51–75] vs. 53 [IQR, 33–75] years; *p* < 0.001) and had higher CRP (6 [IQR, 1–30] vs. 2 [IQR, 1–10] mg/L, *p* = 0.002) and creatinine levels (87 [IQR, 74–109] vs. 77 [IQR, 67–88] µmol/L, *p* < 0.001) (Table 1). Furthermore, they had a higher prevalence of pulmonary hypertension (8.7% vs. 1.2%; *p* < 0.001), atrial fibrillation (15.9% vs. 5.8%; *p* < 0.001), myocardial infarction (39.9% vs. 14.3%; *p* < 0.001), diabetes (27.5% vs. 8.9%; *p* < 0.001), and heart failure (42.8% vs. 8.1%, *p* < 0.001).

### 3.2. Association of Cardiac MRI and Echocardiography Imaging Parameters with NT-proBNP

The patients with elevated NT-proBNP had a higher left ventricular end-diastolic volume index (92 [IQR, 72–112] vs. 81 [IQR, 70–94] mL/m^2^; *p* < 0.001), a lower left ventricular ejection fraction (50 [IQR, 34–62] vs. 60 [IQR, 56–64] %; *p* < 0.001), and a higher left ventricular mass index (77 [IQR, 62–93] vs. 67 [IQR, 57–77] g/m^2^; *p* < 0.001) in the cardiac MRI (Table 2). In the echocardiography, the patients with elevated NT-proBNP had a lower E’ mean (7 [IQR, 5–9] vs. 9 [IQR, 6–12]; *p* < 0.001), higher estimated left ventricular filling pressures E/E’ (11 [IQR, 7–15] vs. 8 [IQR, 7–10]; *p* < 0.001), and a larger LAVI (35 [IQR, 24–43] vs. 26 [IQR, 20–33] ml/m^2^; *p* < 0.001). Additionally, the right ventricular global longitudinal strain (GLS) was lower in the patients with elevated NT-proBNP (14 [IQR, 10–19] vs. 18 [IQR, 14–21] %; *p* < 0.001), and the RV/RA gradients were higher (23 [IQR, 17–33] vs. 20 [IQR, 9–25] mmHg; *p* < 0.001).

### 3.3. Association of Liver T1 and T2 with NT-proBNP

The liver T1 was significantly longer in patients with elevated NT-proBNP when measured in both the right lobe (670 ms [IQR, 648–708; n = 69] vs. 596 ms [IQR, 568–628; n = 23]; *p* < 0.001) and the caudate lobe (664 ms [IQR, 640–708; n = 69] vs. 598 ms [IQR, 578–633; n = 23]; *p* < 0.001) (Table 3). A trend towards higher T1 values was observed in the left lobe (571 ms [IQR, 536–631; n = 190] vs. 568 ms [IQR, 530–606; n = 115]; *p* = 0.068) and liver dome (590 ms [IQR, 540–629; n = 52] vs. 560 ms [IQR, 533–595; n = 19]; *p* = 0.1) (Table 3). No significant difference was found in the liver T2 between the two groups.

**Figure 3:**
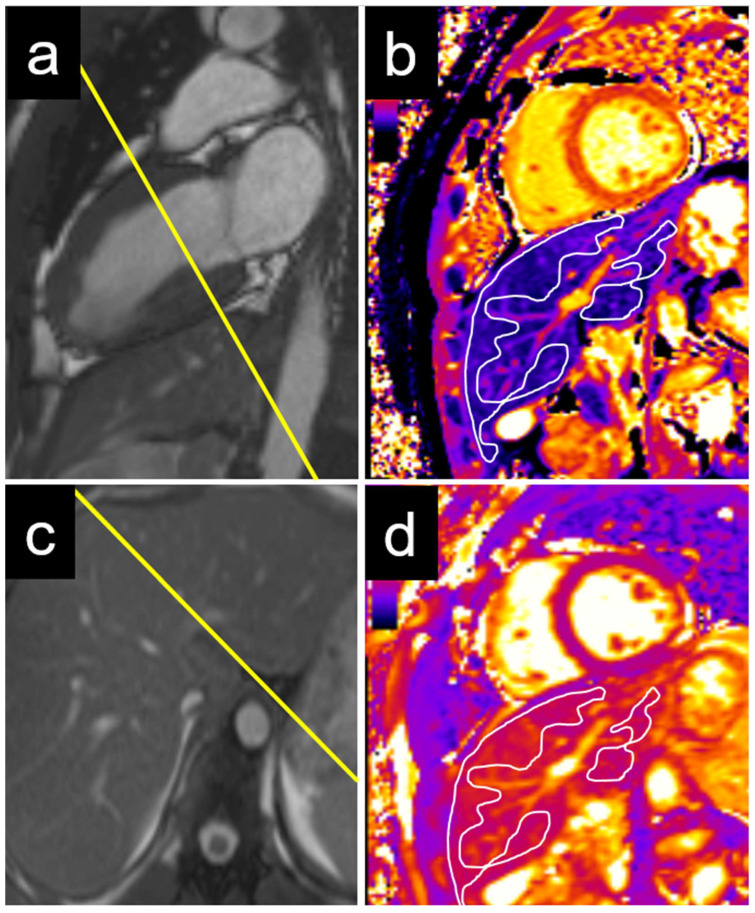
Example of a 22-year-old male patient with normal NT-proBNP. The measurements were performed on a basal short axis view (**a**) in the caudate and right liver lobe T1 (**c**). The liver T1 was 588 ms in the right liver lobe and 563 ms in the caudate lobe (**b**). The liver T2 was 50 ms in the right liver lobe and 47 ms in the caudate lobe (**d**). NT-proBNP = N-terminal pro b-type natriuretic peptide.

**Figure 4:**
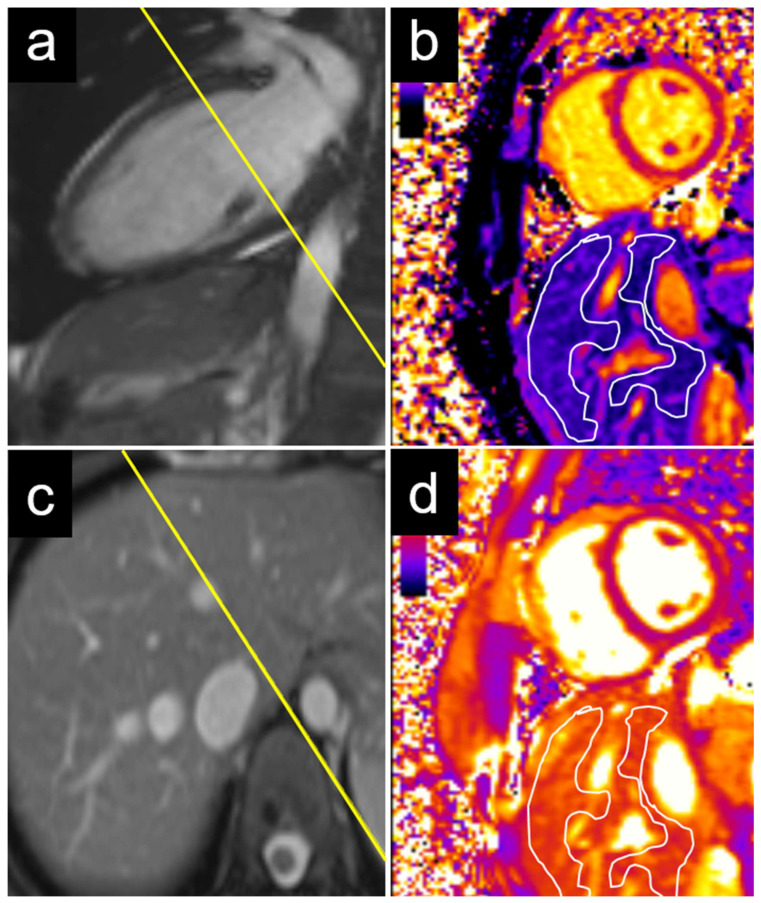
Example of a 29-year-old female patient with elevated NT-proBNP. The measurements were performed on a basal short axis view (**a**) in the caudate and right liver lobes (**c**). The liver T1 was 685 ms in the right liver lobe and 543 ms in the caudate lobe (**b**). The liver T2 was 61 ms in the right liver lobe and 59 ms in the caudate lobe (**d**). NT-proBNP = N-terminal pro b-type natriuretic peptide.

An echocardiographic evaluation revealed similar results, with significant differences in the T1 values between patients with elevated and non-elevated left ventricular filling pressures in the caudate and right liver lobes, but not in the left lobe or liver dome (Table 3). Examples of the liver T1 and T2 measurements are provided for a patient with normal NT-proBNP levels (Figure 3) and for a patient with elevated NT-proBNP levels (Figure 4).

### 3.4. Predictive Value of Liver T1 to Determine Elevated NT-proBNP

In the univariate logistic regression analysis, the liver T1 was associated with elevated NT-proBNP when measured in the caudate (OR 1.016 [95% CI: 1.007, 1.025]; *p* = 0.001) and right liver lobes (OR 1.015 [95% CI: 1.007, 1.023]; *p* < 0.001; Table 4). Additionally, age (OR 1.027 [95% CI: 1.015, 1.039]; *p* < 0.001), LVEF (OR 0.938 [95% CI: 0.921, 0.955]; *p* < 0.001), and atrial fibrillation (OR 3.085 [95% CI: 1.544, 6.166]; *p* = 0.001) were associated with the elevation of NT-proBNP, but not sex, BMI, or creatinine.

The liver T1 remained independently associated with elevated NT-proBNP levels in the multivariate logistic regression model, including age, LVEF, and the presence of atrial fibrillation as potential confounders, when measured in the caudate lobe (OR 1.013 [95% CI: 1.004, 1.023]; *p* = 0.005; Table 4, model 1) and in the right liver lobe (OR 1.012 [95% CI: 1.003, 1.021]; *p* = 0.009; Table 4, model 2).

## 4. Discussion

This study aimed to evaluate the association of congestive heart failure with magnetic resonance liver T1 mapping as a non-invasive imaging surrogate. The liver T1 was significantly increased in patients with elevated NT-proBNP and elevated LV filling pressure evaluated by echocardiography, when measured in the right liver lobe and the caudate lobe. These measurements were obtained from standard cardiac MRI T1 mapping acquisitions on a basal short axis view. However, the liver T1 in the left liver lobe, as obtained from the mid-ventricular short axis view did not show any significant association with heart failure.

This observation may be explained by a hydrostatic effect, since liver congestion due to heart failure is more pronounced in the dependent right liver lobe and caudate lobe, compared to the more central position of the left liver lobe directly adjacent to the heart below the diaphragm.

In accordance with the existing literature, the liver T1 was increased in congestive liver disease, mediated by sinusoidal dilatation and fluid accumulation in the interstitial space [24]. Similarly, results from the Multi-Ethnic Study of Atherosclerosis showed an association between an increased liver T1 and a history of atrial fibrillation, heart failure, and coronary heart disease [27]. Another study showed an association between heart failure and congestive liver disease based on liver stiffness measurements using transient elastography [22]. Recently, an independent predictive value of the liver T1 regarding cardiovascular mortality and morbidity was shown, without closer differentiation of the liver segments [28]. The current findings are in line with those prior studies. However, the current results indicate that an even better association of liver T1 and congestive heart failure is observed when the liver T1 is measured in the caudate or right liver lobe. These findings are supported by observations of patients with Fontan-associated liver disease, showing a large heterogeneity of liver congestion with a higher vulnerability of the caudate and right liver lobes [29,30]. Furthermore, the most cranial parts of the liver, namely, the liver dome and the cranial parts of liver segment II, are prone to partial volume and the susceptibility effects of the air in the adjacent basal parts of the lungs [31].

Another interesting aspect is the correlation between the right heart function and liver T1. A recent study by Ide et al. reported no significant difference regarding the liver T1 in patients with compared to patients without RV enlargement but observed a moderate positive correlation between the RVEDVi and T1 values of the right liver lobe in patients with an enlarged RV in a cohort of congenital heart disease patients [30]. In contrast, our findings demonstrate a significantly lower RVEF in patients with elevated NT-proBNP levels compared to those with normal NT-proBNP levels, despite there being no significant difference in RV volumes between the two groups. These discrepancies may stem from differences in the study populations; notably, patients with severe CHD were excluded from the current analysis, and most participants had indexed RV volumes within the normal range.

However, subsequent studies are warranted to externally validate the current findings and to investigate whether T1 measurements in the caudate and right liver lobes—measured on a cardiac basal short axis view or additional transversal T1 mapping acquisition of the liver—will show a higher association with adverse outcomes than measurements in the left liver lobe on a cardiac midventricular short axis view. In addition, the liver T1 should be investigated in different clinical applications, including, but not limited to, patients with atrial fibrillation, myocarditis, coronary artery disease, metabolic syndrome, and heart failure with a preserved ejection fraction.

The liver T1 is a promising supplemental imaging biomarker for heart failure, and its prognostic significance warrants further investigation in future studies. In this study, the liver T1 demonstrated a correlation with NT-proBNP, a marker of cardiac wall stress frequently related to elevated left ventricular filling pressures. Consequently, the liver T1 may provide valuable additional insights into assessing the actual volume status in patients with heart failure with a reduced ejection fraction (HFrEF) and contribute to the characterization and prognostication of patients with heart failure with a preserved ejection fraction (HFpEF). Further studies should explore these associations and their prognostic significance and implications, including the relevance of an elevated T1 in patients with isolated right heart failure, moderate-to-severe tricuspid regurgitation, and pulmonary hypertension. Based on these findings, the liver T1 measurement in CMR may offer valuable insights for clinicians and potentially influence patient management by facilitating the early diagnosis and timely initiation of heart failure treatment. Conversely, T1 mapping in dedicated liver MRI may provide important information about a patient’s cardiopulmonary status, which is often linked to acute and chronic liver failure, including portopulmonary hypertension. However, the liver T1 should be carefully interpreted in conjunction with other parametric MR sequences to account for potential confounding factors such as liver steatosis, fibrosis, and inflammation.

This study has several limitations. First, this was a retrospective study and the NT-proBNP and echocardiography parameters were not available on the day of the cardiac MRI exams in all the patients. However, the association of an elevated liver T1 and elevated filling pressures was independently confirmed in a separate analysis of a subset of 166 patients with echocardiography. A second limitation is the site-specific character of the results. As mentioned in earlier studies, the T1-weighted mapping values vary between different institutions and scanner vendors [32,33,34]. To allow comparability between different patients, standardized protocols are pertinent to derive the institution-specific reference ranges. Third, NT-proBNP is primarily renally cleared and patients with kidney failure could present with high NT-proBNP levels not linked to heart failure [35]. However, the logistic regression analysis showed no significant association between the serum creatinine and NT-proBNP levels. Finally, no clinical outcome variables such as adverse cardiac events or death by cardiovascular cause were analyzed, since this was not the primary scope of this study and the follow-up intervals would have not been sufficient.

In conclusion, an elevated T1 in the caudate and right liver lobes with cardiac MRI was independently associated with congestive heart failure and performed better than the T1 in the left liver lobe. Dedicated separate liver T1 mapping acquisition may further improve the presented results. If external validation in independent cohorts reveals similar results, the liver T1 may improve patient characterization and the quantification of congestive heart failure with cardiac MRI.

## Figures and Tables

**Table 1:** Patient characteristics.

Characteristic	Total [n = 397]	Normal NT-proBNP [n = 259]	Elevated NT-proBNP ^a^ [n = 138]	*p*-Value ^b^
Age (years)	56 [37–69]	53 [33–75]	61 [51–75]	<0.001
Females (n, %)	127 [32.0%]	89 [34.4%]	40 [29.0%]	0.313
BMI (kg/m^2^)	25 [23–28]	25 [22–28]	25 [23–29]	0.532
Arterial hypertension (n, %)	196 [49.4%]	126 [48.6%]	70 [50.7%]	0.752
LVEF (%)	59 [50–63]	60 [56–64]	50 [34–62]	<0.001
Pulmonary hypertension (n, %)	15 [3.8%]	3 [1.2%]	12 [8.7%]	<0.001
Atrial fibrillation (n, %)	37 [9.3%]	15 [5.8%]	22 [15.9%]	0.002
Myocardial infarction (n, %)	92 [23.3%]	37 [14.3%]	55 [39.9%]	<0.001
Diabetes (n, %)	62 [15.5%]	23 [8.9%]	38 [27.5%]	<0.001
ASAT (U/L)	31 [23–51]	28 [21–44]	33 [24–55]	0.131
ALAT (U/L)	30 [21–60]	28 [20–57]	32 [21–68]	0.323
Albumin (g/L)	35 [30–39]	36 [32–41]	35 [29–38]	0.034
CRP (mg/L)	4 [1–19]	2 [1–10]	6 [1–30]	0.002
Creatinine (µmol/L)	81 [68–98]	77 [67–88]	87 [74–109]	<0.001
Hematocrit	0.39 [0.36–0.42]	0.40 [0.36–0.42]	0.39 [0.35–0.42]	0.453

Continuous parameters as median with interquartile range, categorical parameters as absolute numbers (percentages). BMI = body mass index, LVEF = left ventricular ejection fraction. ^a^ Cutoff value of 125 pg/mL. ^b^ Mann–Whitney U or Fisher’s exact test, as appropriate.

**Table 2:** Association of cardiac MRI and echocardiography imaging parameters with NT-proBNP.

Parameter	All Patients [n = 397]	Normal NT-proBNP [n = 259]	Elevated NT-proBNP [n = 138]	*p*-Value
CMR				
LV EDV index (ml/m^2^)	83 [71–99]	81 [70–94]	92 [72–112]	<0.001
LV ESV index (ml/m^2^)	34 [27–47]	32 [26–39]	41 [30–70]	<0.001
LV mass index (g/m^2^)	70 [59–83]	67 [57–77]	77 [62–93]	<0.001
LVEF (%)	59 [50–63]	60 [56–64]	50 [34–62]	<0.001
RV EDV index (ml/m^2^)	78 [64–91]	78 [67–88]	77 [54–97]	0.629
RV ESV index (ml/m^2^)	31 [24–40]	31 [24–37]	32 [23–50]	0.171
RVEF (%)	60 [54–64]	60 [56–65]	57 [48–63]	<0.001
Echocardiography	All patients [n = 253]	Normal NT-proBNP [n = 113]	Elevated NT-proBNP [n = 140]	*p*-value
LAVI (ml/m^2^)	30 [23–37]	26 [20–33]	35 [24–43]	<0.001
E’ mean	8 [6–11]	9 [6–12]	7 [5–9]	<0.001
E/E’ mean	9 [7–12]	8 [7–10]	11 [7–15]	<0.001
RV/RA (mmHg)	21 [14–27]	20 [9–25]	23 [17–33]	0.011
RAVI (ml/m^2^)	26 [20–35]	25 [20–31]	29 [19–38]	0.181
GLS RV (%)	16 [13–20]	18 [14–21]	14 [10–19]	0.001
VC diameter insp. (mm)	8 [5–12]	8 [6–12]	7 [5–13]	0.587
VC diameter exp. (mm)	18 [14–21]	18 [14–21]	17 [14–21]	0.866

Median values are shown with the upper and lower interquartile range in parentheses. The *p*-values were calculated using the Mann–Whitney U test. Cardiac MRI = cardiac magnetic resonance imaging; EDV = end-diastolic volume; ESV = end-systolic volume; GLS = global longitudinal strain; LAVI = left atrium volume index; LV = left ventricle; LVEF = left ventricular ejection fraction; RA = right atrium; RAVI = right atrium volume index; RV = right ventricle; and VC = vena cava.

**Table 3:** Association of hepatic MR relaxometry with determinants of heart failure.

Parameter	Normal NT-proBNP [n = 259]	Elevated NT-proBNP [n = 138]	*p*-Value
T1 relaxation time (ms)			
Left lobe	568 [530–606]	571 [536–631]	0.068
Liver dome	560 [533–595]	590 [540–629]	0.1
Caudate lobe	598 [578–633]	664 [640–708]	<0.001
Right lobe	596 [568–628]	670 [648–708]	<0.001
T2 relaxation time (ms)			
Left lobe	50 [47–54]	50 [46–54]	0.525
Liver dome	53 [48–55]	52 [50–55]	0.920
Caudate lobe	52 [49–56]	54 [47–58]	0.572
Right lobe	52 [48–57]	54 [50–60]	0.228
	Normal LV filling pressure [n = 104]	Elevated LV filling pressure [n = 62]	*p*-value
T1 relaxation time (ms)			
Left lobe	570 [532–616]	583 [536–617]	0.478
Liver dome	588 [536–624]	565 [495–589]	0.288
Caudate lobe	596 [572–621]	654 [615–660]	0.007
Right lobe	596 [565–625]	639 [615–655]	0.014

Median values are shown with the upper and lower interquartile range in parentheses. The *p*-values were calculated using the Mann–Whitney U test.

**Table 4:** Results of logistic regression analysis.

VARIABLE	ODDS RATIO	95% CONFIDENCE INTERVAL	*p*-VALUE
Hepatic T1 time			
Left lobe	1.003	1.000–1.006	0.056
Liver dome	1.007	0.999–1.016	0.098
Caudate lobe	1.016	1.007–1.025	0.001
Right lobe	1.015	1.007–1.023	<0.001
Age	1.027	1.015–1.039	<0.001
LVEF	0.938	0.921–0.955	<0.001
BMI	1.025	0.990–1.061	0.158
Gender	1.283	0.819–2.008	0.276
Atrial fibrillation	3.085	1.544–6.166	0.001
Creatinine	1.003	0.998–1.008	0.241
Multivariate Model 1			
T1 mapping caudate lobe	1.013	1.004–1.023	0.005
Age	1.004	0.968–1.042	0.829
Atrial fibrillation	1.867	0.112–31.057	0.663
LVEF	0.958	0.915–1.003	0.067
Multivariate Model 2			
T1 mapping right lobe	1.012	1.003–1.021	0.009
Age	1.007	0.972–1.044	0.692
Atrial fibrillation	1.598	0.090–28.282	0.749
LVEF	0.965	0.922–1.009	0.119

Univariate logistic regression analysis of the predictive value of liver T1, age, LVEF, BMI, gender, atrial fibrillation, and creatinine to determine elevated NT-proBNP. Multivariate logistic regression analysis of the predictive value of liver T1 in the caudate lobe (model 1) and the right liver lobe (model 2) to determine elevated NT-proBNP, when corrected for age, atrial fibrillation, and LVEF. LVEF = left ventricular ejection fraction; BMI = body mass index; and NT-proBNP = N-terminal pro b-type natriuretic peptide.

## Data Availability

The datasets generated and/or analyzed during this current study are available from the corresponding author on reasonable request as they were exported from the local PACS system and are not part of an openly available repository.

## Supplementary Materials

The following supporting information can be downloaded at: https://www.mdpi.com/article/10.3390/diagnostics15060779/s1, Table S1: CMR indications.

## Abbreviations

ALAT	Alanine aminotransferase
ASAT	Aspartate aminotransferase
BMI	Body mass index
CMR	Cardiac magnetic resonance
CLD	Chronic liver disease
TDI	Tissue Doppler imaging
E/A	Mitral inflow early-to-late diastolic flow ratio
E’	Peak early diastolic tissue velocity of mitral annulus
ECV	Extracellular volume
EF	Ejection fraction
GLS	Global longitudinal strain
DCM	Dilated cardiomyopathy
LAVI	Left atrial volume index
MRI	Magnetic resonance imaging
NT-proBNP	N-terminal pro-Brain natriuretic peptide
NYHA	New York Heart Association
ROI	Region of interest
RV S’	Tissue Doppler peak systolic velocity at RV lateral annulus
TAPSE	Tricuspid annular plane systolic excursion
