# Supplementary material for: The Association of Heart Failure and Liver T1 Mapping in Cardiac Magnetic Resonance Imaging
Source: Diagnostics (Basel). 2025 Mar 20;15(6):779. doi: 10.3390/diagnostics15060779 (PMC11941626; doi:10.3390/diagnostics15060779)
Supplement: Supplementary file 1 [file diagnostics-15-00779-s001.zip › diagnostics-3413839-supplementary.pdf]

Supplementary materials

Table S1: CMR indications

Indication	Number of Patients, n (%)
**Cardiomyopathy**	90 (22.7%)
**Ischemia/Viability**	116 (29.2%)
**Peri-/Myocarditis**	44 (11.1%)
**Valvulopathy / Shunt**	5 (1.3%)
**Congenital heart disease / Aortopathy**	97 (24.4%)
**Tumor / Thrombus**	9 (2.3%)
**Atrial fibrillation**	27 (6.8%)
**Post myocardial infarction / MVO**	9 (2.3%)

Table S1. CMR indications

MVO, microvascular obstruction
